# Effects of Empagliflozin on Symptoms, Physical Limitations, and Quality of Life in Patients Hospitalized for Acute Heart Failure: Results From the EMPULSE Trial

**DOI:** 10.1161/CIRCULATIONAHA.122.059725

**Published:** 2022-04-04

**Authors:** Mikhail N. Kosiborod, Christiane E. Angermann, Sean P. Collins, John R. Teerlink, Piotr Ponikowski, Jan Biegus, Josep Comin-Colet, João Pedro Ferreira, Robert J. Mentz, Michael E. Nassif, Mitchell A. Psotka, Jasper Tromp, Martina Brueckmann, Jonathan P. Blatchford, Afshin Salsali, Adriaan A. Voors

**Affiliations:** Saint Luke’s Mid America Heart Institute, Kansas City, MO (M.N.K., M.E.N.).; School of Medicine, University of Missouri-Kansas City (M.N.K., M.E.N.).; The George Institute for Global Health, University of New South Wales, Sydney, Australia (M.N.K.).; Comprehensive Heart Failure Centre, University and University Hospital of Würzburg, Germany (C.E.A.).; Department of Emergency Medicine, Vanderbilt University Medical Center, Nashville, TN (S.P.C.).; Geriatric Research and Education Clinical Care, Tennessee Valley Healthcare Facility VA Medical Center, Nashville (S.P.C.).; Section of Cardiology, San Francisco Veterans Affairs Medical Center and School of Medicine, University of California San Francisco (J.R.T.).; Institute of Heart Diseases, Medical University, Wroclaw, Poland (P.P., J.B.).; Hospital Universitari de Bellvitge, The Institute of Biomedical Research of Bellvitge (IDIBELL), Barcelona, Spain (J.C.-C.).; Université de Lorraine, Inserm INI-CRCT, CHRU, Nancy, France (J.P.F.).; Cardiovascular Research and Development Center, Department of Surgery and Physiology, Faculty of Medicine of the University of Porto, Portugal (J.P.F.).; Duke Clinical Research Institute and Division of Cardiology, Duke University Medical Center, Durham, NC (R.J.M.).; Inova Heart and Vascular Institute, Falls Church, VA (M.A.P.).; Saw Swee Hock School of Public Health, National University of Singapore (J.T.).; Boehringer Ingelheim International GmbH, Germany (M.B.).; First Department of Medicine, Faculty of Medicine Mannheim, University of Heidelberg, Mannheim, Germany (M.B.).; Elderbrook Solutions GmbH on behalf of Boehringer Ingelheim Pharma GmbH & Co. KG, Biberach, Germany (J.P.B.).; Boehringer Ingelheim Pharmaceuticals Inc, Ridgefield, CT (A.S.).; Faculty of Medicine, Rutgers University, New Brunswick, NJ (A.S.).; University of Groningen, Department of Cardiology, University Medical Center Groningen, The Netherlands (A.A.V.).

**Keywords:** acute heart failure, quality of life, SGLT2 inhibitor

## Abstract

Patients hospitalized for acute heart failure experience poor health status, including a high burden of symptoms and physical limitations, and poor quality of life. SGLT2 (sodium-glucose cotransporter 2) inhibitors improve health status in chronic heart failure, but their effect on these outcomes in acute heart failure is not well characterized. We investigated the effects of the SGLT2 inhibitor empagliflozin on symptoms, physical limitations, and quality of life, using the Kansas City Cardiomyopathy Questionnaire (KCCQ) in the EMPULSE trial (Empagliflozin in Patients Hospitalized With Acute Heart Failure Who Have Been Stabilized).

**Methods:**

Patients hospitalized for acute heart failure were randomized to empagliflozin 10 mg daily or placebo for 90 days. The KCCQ was assessed at randomization and 15, 30, and 90 days. The effects of empagliflozin on the primary end point of clinical benefit (hierarchical composite of all-cause death, heart failure events, and a 5-point or greater difference in KCCQ Total Symptom Score [TSS] change from baseline to 90 days) were examined post hoc across the tertiles of baseline KCCQ-TSS. In prespecified analyses, changes (randomization to day 90) in KCCQ domains, including TSS, physical limitations, quality of life, clinical summary, and overall summary scores were evaluated using a repeated measures model.

**Results:**

In total, 530 patients were randomized (265 each arm). Baseline KCCQ-TSS was low overall (mean [SD], 40.8 [24.0] points). Empagliflozin-treated patients experienced greater clinical benefit across the range of KCCQ-TSS, with no treatment effect heterogeneity (win ratio [95% CIs] from lowest to highest tertile: 1.49 [1.01–2.20], 1.37 [0.94–1.99], and 1.48 [1.00–2.20], respectively; *P* for interaction=0.94). Beneficial effects of empagliflozin on health status were observed as early as 15 days and persisted through 90 days, at which point empagliflozin-treated patients experienced a greater improvement in KCCQ TSS, physical limitations, quality of life, clinical summary, and overall summary (placebo-adjusted mean differences [95% CI]: 4.45 [95% CI, 0.32–8.59], *P*=0.03; 4.80 [95% CI, 0.00–9.61], *P*=0.05; 4.66 [95% CI, 0.32–9.01], *P*=0.04; 4.85 [95% CI, 0.77–8.92], *P*=0.02; and 4.40 points [95% CI, 0.33–8.48], *P*=0.03, respectively).

**Conclusions:**

Initiation of empagliflozin in patients hospitalized for acute heart failure produced clinical benefit regardless of the degree of symptomatic impairment at baseline, and improved symptoms, physical limitations, and quality of life, with benefits seen as early as 15 days and maintained through 90 days.

**Registration:**

URL: https://www.clinicaltrials.gov; Unique identifier: NCT0415775.

**Clinical Perspective**

**What Is New?**

- Patients hospitalized for acute heart failure experience a high burden of symptoms and physical limitations and poor quality of life. In the post hoc and prespecified analyses of EMPULSE trial (Empagliflozin in Patients Hospitalized With Acute Heart Failure Who Have Been Stabilized), we investigated the effects of the SGLT2 (sodium-glucose cotransporter 2) inhibitor empagliflozin on total clinical benefit across the range of symptomatic impairment at baseline and its effect on symptoms, physical limitations, and quality of life, using the Kansas City Cardiomyopathy Questionnaire.
- We found that initiation of empagliflozin in patients hospitalized for acute heart failure produced clinical benefit regardless of the degree of symptomatic impairment at baseline and improved symptoms, physical limitations, and quality of life, with benefits seen as early as 15 days and maintained through 90 days.

**What Are the Clinical Implications?**

- These observations are of clinical relevance because few therapies have been shown to improve symptoms and functional status in the early postdischarge period in patients hospitalized with acute heart failure.
- The findings were consistent across multiple subgroups, extending the health status benefits of SGLT2 inhibitors to patients hospitalized with acute heart failure regardless of ejection fraction, de novo versus chronic decompensated heart failure status, and degree of symptomatic impairment at baseline.

**Editorial, see p 299**

Patients hospitalized for acute heart failure (AHF) are at high risk for cardiovascular death and readmission, and also experience an especially high burden of heart failure (HF)–related symptoms and physical limitations (PLS).^1–3^ Accordingly, improving health status (symptoms, functional status, and quality of life [QoL]) is a key goal of HF management in this vulnerable group. To date, there has been a lack of therapies with a compelling benefit on these outcomes in individuals with AHF, highlighting a critical unmet need.

SGLT2 (sodium-glucose cotransporter 2) inhibitors have emerged as a therapeutic option for patients with HF. Several clinical trials have demonstrated that these agents significantly improve symptoms and PLS in ambulatory patients with HF, regardless of ejection fraction.^4–8^ However, whether these effects are also observed in individuals with AHF—the group with greatest symptomatic and functional limitations—remains largely unknown.

In the EMPULSE trial (Empagliflozin in Patients Hospitalized With Acute Heart Failure Who Have Been Stabilized), we previously demonstrated that empagliflozin 10 mg daily, compared with placebo, resulted in a significant clinical benefit (composite of death, HF events, or change in symptom burden) among individuals hospitalized with AHF, regardless of ejection fraction, HF status (de novo versus decompensated chronic HF), or diabetes status.^9,10^ In this report, we sought to address the following 2 key objectives: (1) to evaluate, in post hoc analyses, whether the effects of empagliflozin on the primary end point of total clinical benefit in the EMPULSE trial varied according to the degree of symptomatic impairment at baseline; and (2) to examine, in prespecified analyses, the effect of empagliflozin on the broader range of health status outcomes, as measured by the various domains of the Kansas City Cardiomyopathy Questionnaire (KCCQ)—a validated, self-administered instrument that quantifies HF-related symptoms, function, and QoL,^11^ as well as the time course of these effects.

## Methods

EMPULSE (URL: https://www.clinicaltrials.gov; Unique identifier: NCT0415775) was a randomized, double-blind, placebo-controlled trial in patients with AHF (both de novo and decompensated chronic HF, with reduced or preserved ejection fraction, and with or without type 2 diabetes) that evaluated the efficacy and safety of empagliflozin 10 mg once daily, compared with placebo, when added to standard care. The design, baseline characteristics, and primary results of the trial have been published.^9^ The Ethics Committee of each of the 118 participating institutions (in 15 countries) approved the protocol, and all patients gave written informed consent. Boehringer Ingelheim was responsible for data collection and storage. The academic members of the executive committee provided an independent interpretation of the results. The authors made the decision to submit the article for publication, and assume full responsibility for the accuracy and completeness of the data.

### Study Patients

Men and women aged ≥18 years (or above the age of legal consent according to local legislation) were eligible if they were hospitalized with a primary diagnosis of AHF with dyspnea on exertion or at rest, and had at least 2 of the following: congestion on chest radiograph, rales on chest auscultation, clinically relevant edema, or an elevated jugular venous pressure. Participants were randomized after at least 24 hours and no later than 5 days after admission, as early as possible after stabilization, and while still in the hospital. Stabilization criteria were a systolic blood pressure of at least 100 mm Hg; no inotropic support for at least 24 hours; no symptoms of hypotension; no increase in the intravenous diuretic dose and no intravenous vasodilators in the 6 hours before randomization. Patients were required to have an NT-proBNP (N-terminal pro-B-type natriuretic peptide) concentration of at least 1600 pg/mL or a BNP (B-type natriuretic peptide) concentration of at least 400 pg/mL (NT-proBNP or BNP of at least 2400 pg/mL or 600 pg/mL, respectively, if atrial fibrillation was present) and have received at least 40 mg (20 mg for Japanese patients) of intravenous furosemide or equivalent. Key exclusion criteria included cardiogenic shock; pulmonary embolism, cerebrovascular accident, or acute myocardial infarction as the primary trigger for the current hospitalization or in the preceding 90 days; current or expected cardiac transplantation, left ventricular assist device, or inotropic support; an estimated glomerular filtration rate <20 mL/min/1.73m^2^ or requiring dialysis; and previous ketoacidosis. A full list of exclusion criteria is provided in the design article.^10^

### Study Procedures

After the provision of informed consent, patients were screened, and if eligible, randomized to empagliflozin 10 mg daily or matching placebo. Efficacy and safety parameters were assessed during follow-up visits at 3, 5, 15, 30, and 90 days after randomization. During the onsite visits at 15, 30, and 90 days, patients’ health status was assessed using the KCCQ. Because of the ongoing COVID-19 pandemic, phone or home visits were allowed if patients were unable to attend the study visit in person.

### Primary Outcome

The primary outcome was clinical benefit at 90 days, defined as a hierarchical composite end point of time to all-cause death, the number of HF events (defined as HF hospitalizations, urgent HF visits, and unplanned outpatient HF visits), time to first HF event, and a 5-point or greater difference in change from baseline in KCCQ Total Symptom Score (TSS) after 90 days of treatment.

### Kansas City Cardiomyopathy Questionnaire

The KCCQ was completed electronically by patients, without assistance by site study staff (as validated), and evaluated at randomization, 15, 30, and 90 days. The KCCQ is a 23-item, self-administered disease-specific instrument that quantifies symptoms (frequency, severity, and recent change), physical function, QoL, and social function over the previous 2 weeks. KCCQ-TSS, which was a prespecified secondary outcome, quantifies the symptom frequency and severity. Additional domains included in this analysis were KCCQ PLS and QoL scores, as well as clinical summary score (CSS), which incorporates the physical function and symptoms domains, and KCCQ overall summary score (OSS), which is derived from all domains (TSS, physical function, QoL, and social function). For each domain, the validity, reproducibility, responsiveness, and interpretability have been independently established.^11^ Scores are transformed to a range of 0 to 100, in which higher scores reflect better health status.

### Statistical Analysis

In a post hoc analysis, patients were divided into 3 subgroups, on the basis of the tertiles of baseline KCCQ-TSS (which was the KCCQ domain prespecified as a component of the primary, as well as a secondary end point): (1) <27.1, (2) ≥27.1 to <52.1, and (3) ≥52.1 points. Baseline characteristics were summarized as means and SDs, medians, and interquartile ranges, or percentages. Ordinal regression likelihood ratio tests were used to compare trends across tertile categories of KCCQ-TSS at baseline.

To evaluate the effects of empagliflozin versus placebo on the primary hierarchical composite end point of clinical benefit across the KCCQ-TSS tertiles, we conducted a post hoc analysis comparing patients randomized to empagliflozin with those randomized to placebo, within tertiles of baseline KCCQ-TSS. Each comparison of 2 patients followed the hierarchy of comparing time to death, number of HF events, time to HF event, or a 5-point or greater difference in change from baseline in the KCCQ-TSS at day 90, until conclusion of a win or loss or otherwise concluding by a tie, as previously described.^9^ We calculated the win ratio as the number of wins in the empagliflozin group divided by the number of losses. The treatment effect by baseline KCCQ-TSS tertile interaction was tested using Cochran’s Q statistic (inverse variance-weighting approach). A multiple imputation approach, according to whether patients were on treatment or off treatment, was used to impute missing data for the KCCQ-TSS, as previously described.^9^

We analyzed the differences between treatment groups in mean KCCQ-TSS, PLS, QoL, CSS, and OSS at days 15, 30, and 90 using a mixed effects model for repeated measures adjusted for HF status and baseline score by visit interaction; KCCQ-TSS at 90 days was a prespecified secondary end point, and the rest were prespecified exploratory end points. In addition, we performed a post hoc analysis evaluating whether the effects of empagliflozin versus placebo on change in KCCQ-TSS from baseline to 90 days differed across multiple subgroups on the basis of patients’ demographic and clinical characteristics at baseline.

We conducted responder analyses examining proportions of patients with a deterioration (≥5 point worsening), and clinically important improvements in KCCQ-TSS at 90 days, using established, clinically meaningful thresholds for KCCQ (≥5 point [at least small], ≥10 point [moderate], and ≥20 point [large] change) for all responder analyses. Among these outcomes, the proportion of patients with a ≥10 point improvement in KCCQ-TSS was a prespecified secondary, whereas ≥5 point and ≥20 point improvements were prespecified exploratory end points, and ≥5 point worsening end point was analyzed post hoc. The proportion of responders was compared between those treated with empagliflozin versus placebo using multiple imputation to account for missing KCCQ values. Odds ratios to estimate differences between treatment groups, and their corresponding 95% CIs and 2-sided *P* values, were estimated from logistic regression models (which included treatment group, stratification variable [de novo versus decompensated chronic HF], and baseline KCCQ-TSS values). All analyses were performed with SAS software, version 9.3 or higher (SAS Institute). *P* values of 0.05 were considered statistically significant, and were not adjusted for multiple comparisons.

### Data Sharing Statement

The data that support the findings of this study are available on reasonable request. To ensure independent interpretation of clinical study results and enable authors to fulfill their role and obligations under the International Committee of Medical Journal Editors criteria, Boehringer Ingelheim grants all external authors access to relevant clinical study data. In adherence with the Boehringer Ingelheim Policy on Transparency and Publication of Clinical Study Data, scientific and medical researchers can request access to clinical study data after publication of the primary article in a peer-reviewed journal, regulatory activities are complete, and other criteria are met. Researchers should use the https://vivli.org/ link to request access to study data and visit https://www.mystudywindow.com/msw/datasharing for further information.

## Results

Overall, 530 patients were randomized (265 each arm); 526 patients (99.2% of the overall trial population) had KCCQ data available at baseline. Of these, 473 patients (89.2% of the overall trial population) had KCCQ evaluated at 15 days; 471 (88.9% of the overall trial population) had KCCQ evaluated at 30 days; and 451 (85.1% of the overall trial population) had KCCQ evaluated at 90 days. The proportions of patients with missing KCCQ values were similar in the empagliflozin and placebo groups at 15, 30, and 90 days (12.1% versus 9.4%, 10.6% versus 11.7%, and 13.2% versus 16.6%, respectively). The number and proportion of patients in the KCCQ-TSS tertiles are shown in the Table.

**Table.**
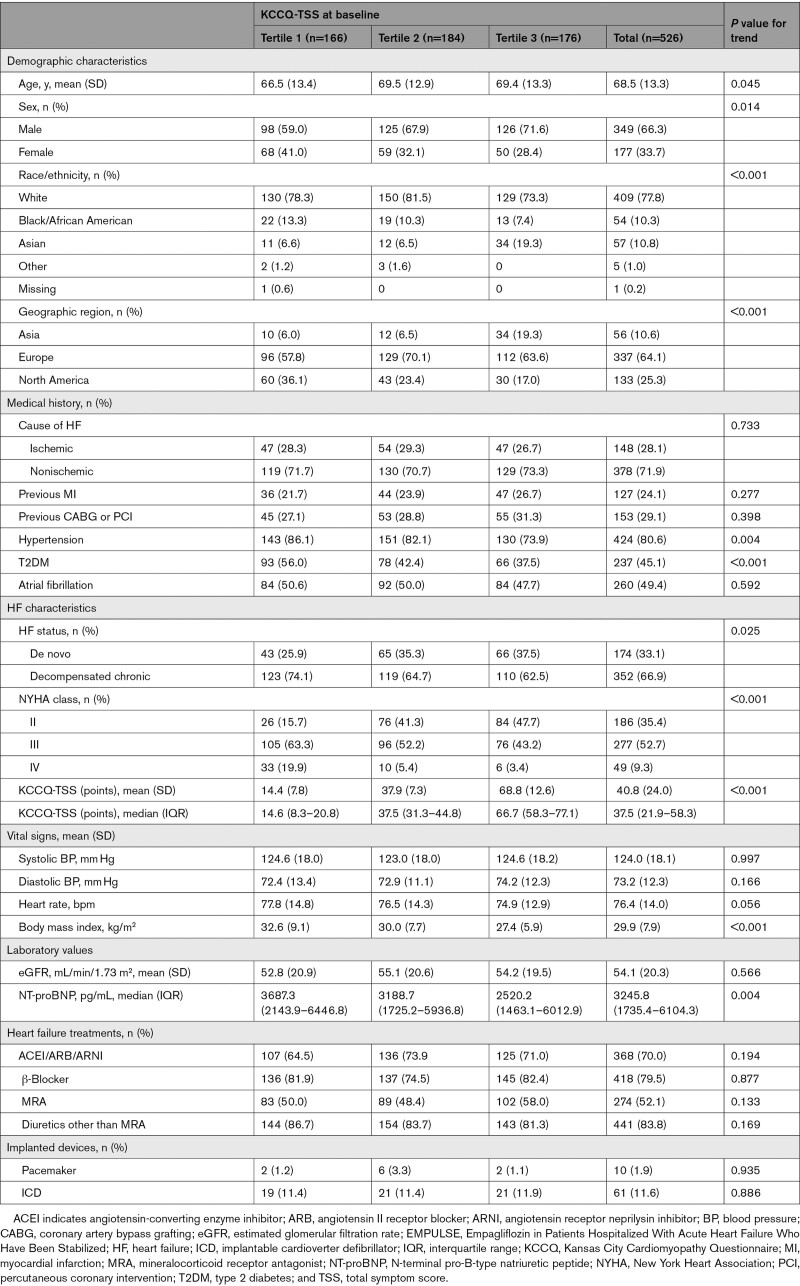
Baseline Characteristics of the EMPULSE Study Population by Tertiles of KCCQ

Compared with participants with higher KCCQ-TSS scores at baseline, those with lower scores were younger; more often women, Black or African American, enrolled in North America, with a history of hypertension and type 2 diabetes; had higher New York Heart Association class, body mass index, and NT-proBNP; and were more likely to have chronic decompensated versus de novo AHF (Table).

### Clinical Outcomes

**Figure 1.**
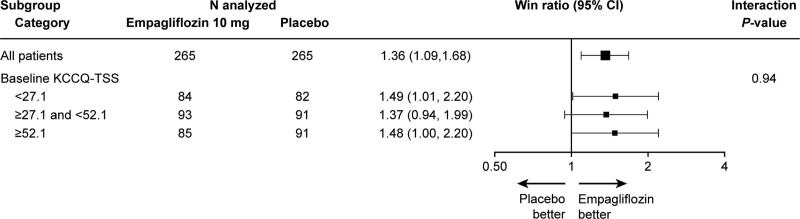
**Effects of empagliflozin vs placebo on the primary hierarchical composite end point of clinical benefit across tertiles of KCCQ-TSS.** KCCQ-TSS indicates Kansas City Cardiomyopathy Questionnaire-Total Symptom Score.

The effects of empagliflozin on the primary hierarchical end point of clinical benefit stratified by KCCQ-TSS tertiles are summarized in Figure 1. Patients treated with empagliflozin experienced greater clinical benefit across the range of KCCQ-TSS, with no evidence of treatment effect heterogeneity (win ratio [95% CI] from lowest to highest tertile: 1.49 [95% CI, 1.01–2.20], 1.37 [95% CI, 0.94–1.99], and 1.48 [95% CI, 1.00–2.20], respectively; *P* for interaction=0.94; Figure 1).

### Health Status Outcomes

**Figure 2.**
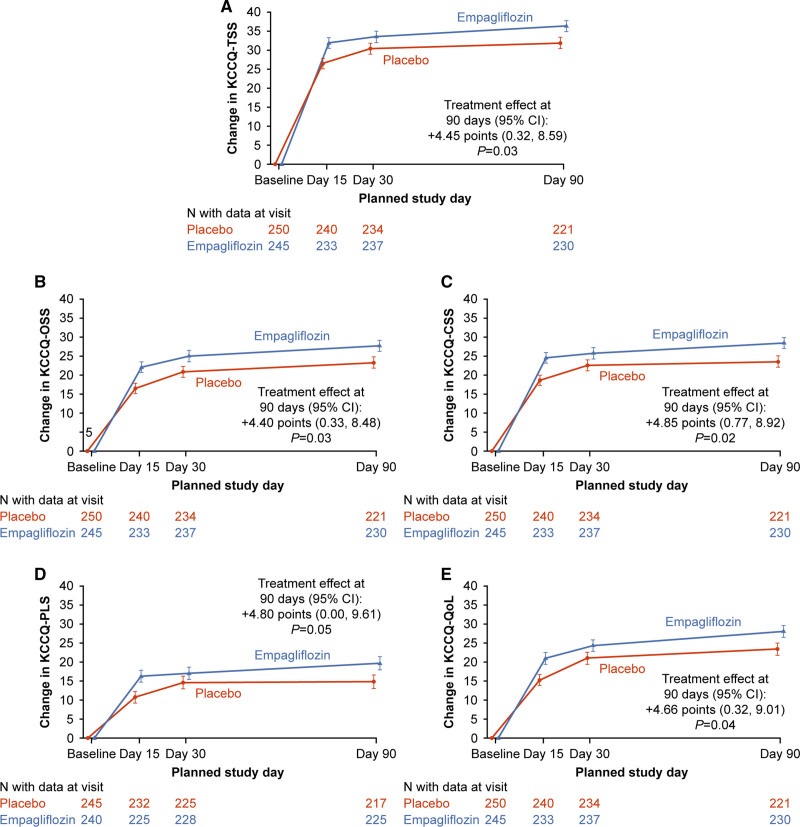
**Effects of empagliflozin vs placebo on change in KCCQ domains over time. A**, TSS. **B**, OSS. **C**, CSS. **D**, PLS. **E**, QoL. The data are presented as adjusted least-square means ± SE. CSS indicates clinical summary score; KCCQ, Kansas City Cardiomyopathy Questionnaire; OSS, overall summary score; PLS, physical limitations score; QoL, quality of life; and TSS, total symptom score.

**Figure 3.**
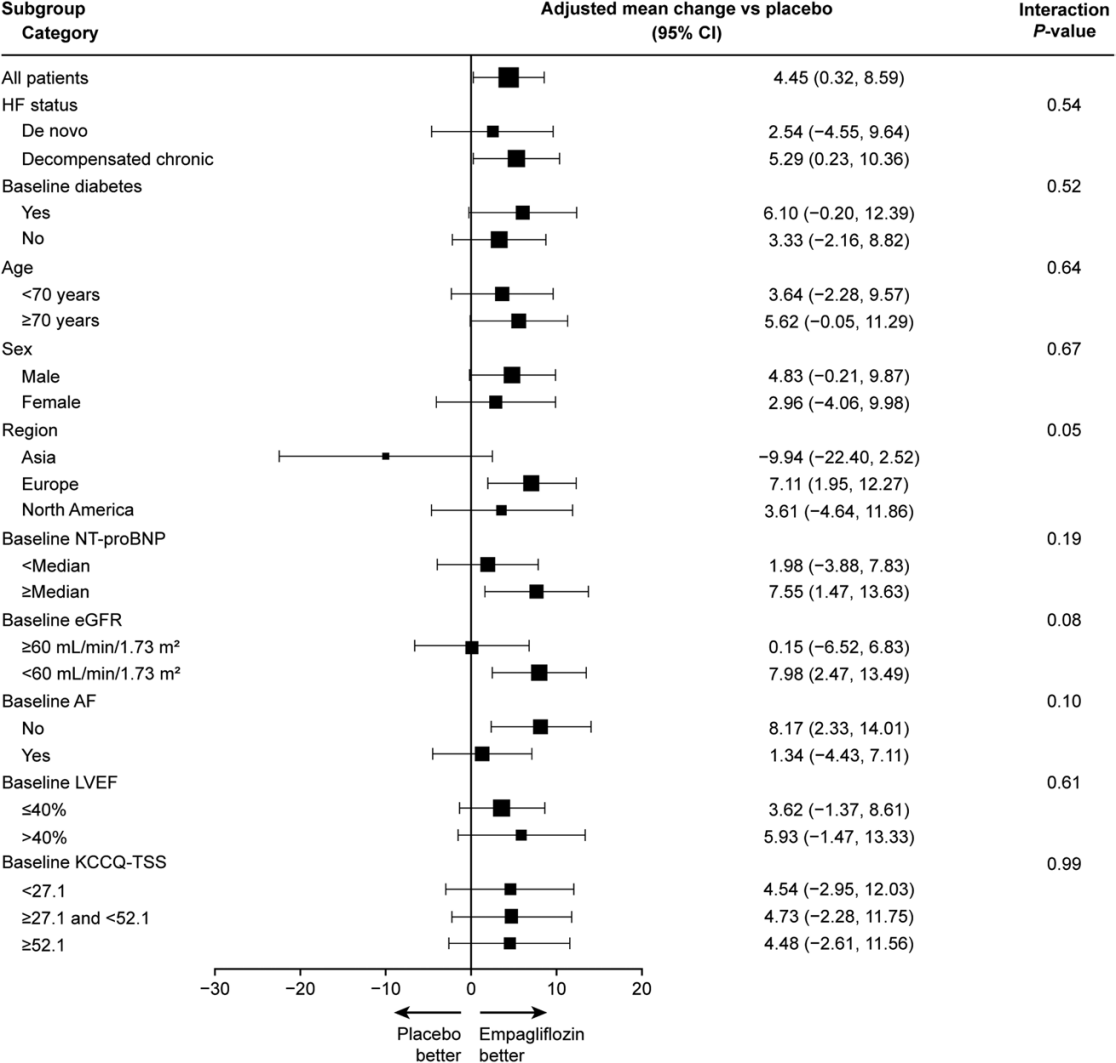
**Effects of empagliflozin vs placebo on KCCQ-TSS at day 90 across prespecified subgroups.** AF indicates atrial fibrillation; eGFR, estimated glomerular filtration rate; HF, heart failure; KCCQ-TSS, Kansas City Cardiomyopathy Questionnaire-Total Symptom Score; LVEF, left ventricular ejection fraction; and NT-proBNP, N-terminal pro-B-type natriuretic peptide.

Baseline KCCQ-TSS was low overall (mean [SD], 40.8 [24.0] points), and improved substantially by 90 days (mean [95% CI] change in empagliflozin group, +36.2 [95% CI, 33.3–39.1]; placebo group, +31.7 [95% CI, 28.8–34.7]). The mean changes in KCCQ-TSS, PLS, QoL, CSS, and OSS over time are presented in Figure 2A, 2B, 2C, 2D, and 2E, respectively. Empagliflozin-treated patients had a greater improvement in KCCQ-TSS, PLS, QoL, CSS, and OSS at day 90 (placebo-adjusted mean differences [95% CI]: 4.45 [95% CI, 0.32–8.59], *P*=0.03; 4.80 [95% CI, 0.00–9.61], *P*=0.05; 4.66 [95% CI, 0.32–9.01], *P*=0.04; 4.85 [95% CI, 0.77–8.92], *P*=0.02; and 4.40 [95% CI, 0.33–8.48], *P*=0.03, respectively), with significant benefits already present as early as 15 days (eg, for KCCQ-TSS, placebo-adjusted mean difference, 5.35 [95% CI, 1.51–9.19] points; *P*<0.01) and maintained through 90 days. The results in terms of KCCQ-TSS at 90 days for empagliflozin versus placebo-treated patients were generally consistent across the prespecified demographic and clinical subgroups, including ejection fraction of ≤40 versus >40%, de novo versus chronic decompensated AHF, and degree of symptomatic impairment (measured by tertiles of KCCQ-TSS) at baseline (Figure 3).

The results of the responder analysis at 90 days are shown in Figure S1, and include the proportion of patients treated with empagliflozin versus placebo that had a clinically significant deterioration (≥5 point decline in KCCQ-TSS [8.0% versus 11.7%; odds ratio, 0.67 [95% CI, 0.35–1.30; *P*=0.24); and at least small (87.5% versus 82.4%), moderate (83.1% versus 76.3%), or large (70.4% versus 65.5%) improvements in KCCQ-TSS (corresponding odds ratio [95% CI]: 1.49 [95% CI, 0.84–2.64], *P*=0.17; 1.52 [95% CI, 0.93–2.50], *P*=0.10; 1.22 [95% CI, 0.78–1.89], *P*=0.38); these results did not reach statistical significance.

## Discussion

In this secondary analysis of the EMPULSE trial, we observed that treatment with empagliflozin improved the end point of total clinical benefit among patients hospitalized with AHF to a similar extent across the entire range of KCCQ, indicating that the beneficial effects of empagliflozin on HF outcomes in this patient group are independent of the health status impairment at baseline. Moreover, empagliflozin significantly improved all key KCCQ domains, including TSS, PLS, QoL, CSS, and OSS (which collectively encompass symptoms, physical function, QoL, and social function), with benefits generally consistent regardless of demographic and clinical characteristics, seen as early as 15 days, and maintained through 90 days.

These results expand on the previously reported effects of empagliflozin specifically, and SGLT2 inhibitors overall, on health status, as measured by KCCQ in patients with HF. Several previous trials demonstrated that empagliflozin and dapagliflozin improve symptoms and PLS in outpatients with HF and reduced ejection fraction.^4–6^ More recent trials also show that these agents similarly improve health status in ambulatory patients with HF and preserved ejection fraction.^7,8^ However, until now, the data about the effects of this class on symptoms, physical function, and QoL in patients hospitalized for AHF have been limited. The only trial that previously evaluated a similar patient population is SOLOIST-WHF (Effect of Sotagliflozin on Cardiovascular Events in Patients With Type 2 Diabetes Post Worsening Heart Failure), which enrolled patients with type 2 diabetes hospitalized with or recently discharged after an episode of decompensated HF, and showed favorable effects of sotagliflozin, a mixed SGLT1/2 inhibitor, on the 12-item KCCQ score at 4 months (mean 4.1-point improvement compared with placebo).^12^ Our findings from EMPULSE add to these data in several ways. First, we specifically focused on patients earlier in the course of hospitalization, during a more acute phase. Second, we included patients with de novo AHF, a previously unstudied but important group. Third, our trial enrolled individuals both with and without type 2 diabetes. Fourth, we demonstrated the benefits of SGLT2 inhibition in the immediate postdischarge period, starting as early as 15 days. Last, we extended the analyses to the entire spectrum of HF-related health status by evaluating all key KCCQ domains.

Our results are of clinical importance, because few therapies have been previously shown to improve symptoms and functional status in the early postdischarge period in individuals hospitalized with AHF. To our knowledge, the only other pharmacotherapy shown to have such benefits (aside from SGLT inhibition) is intravenous ferric carboxymaltose, which improved KCCQ-12 OSS by 3.7 points at 12 weeks in the AFFIRM-AHF trial (Study to Compare Ferric Carboxymaltose With Placebo in Patients With Acute Heart Failure and Iron Deficiency).^13^ However, that study specifically focused on individuals with iron deficiency and ejection fraction <50%, and these benefits did not emerge until after the first 2 weeks. Demonstrating health status benefits in this patient population is especially challenging, because the individuals hospitalized for AHF have marked symptomatic and functional impairments at the time of randomization, and because the result of aggressive management during hospitalization and the immediate postdischarge period tend to experience marked improvements in health status, regardless of whether they are assigned to active treatment or placebo. This was the case in EMPULSE, as well as in many other previous trials in AHF.^2,12,13^ Our findings of significant improvements in every domain of KCCQ, seen just after 2 weeks postdischarge, and persisting to 3 months, are, therefore, especially notable.

These observations may also be of potential relevance when considered in the context of the 30-day mortality and readmission metric, which, at least in the United States, is currently used to assess the quality of care for patients hospitalized for AHF (and for which poor performance may lead to financial penalties).^14^ To our knowledge, the early improvement in KCCQ (a well-known predictor of cardiovascular death and HF readmissions^15^) that we observed with empagliflozin at 15 days is the first such observation, and if corroborated by future studies would suggest that initiation of SGLT2 inhibitors during hospitalization for AHF may be a tool for improving the quality of hospital-to-home transitions.

The results of this study should be considered in the context of several potential limitations. First, although KCCQ-TSS was a component of the primary composite end point, and a predefined secondary end point, and prospective assessments of KCCQ domains were prespecified, several of the analyses, including the evaluation of the primary end point by tertiles of baseline KCCQ-TSS, were performed post hoc. Second, as in most trials, some patients had missing health status assessments during follow-up; however, the proportion of participants with missing KCCQ values was similar among those treated with empagliflozin and placebo. Third, the relatively modest sample size of this study did not provide sufficient power for the responder analyses. However, the large spontaneous improvement in KCCQ observed in both the empagliflozin and placebo groups during the immediate postdischarge period (and likely associated with intensification of HF treatment) makes the traditional KCCQ thresholds for responder analyses more methodologically challenging, and less meaningful. Fourth, the follow-up period of 90 days was relatively short. Last, as in all trials, the inclusion and exclusion criteria may limit generalizability.

In conclusion, initiation of empagliflozin in patients hospitalized for AHF produced clinical benefit regardless of the degree of symptomatic impairment at baseline, and improved symptoms, PLS, and QoL, with benefits seen as early as 15 days and maintained through 90 days.

## Article Information

This work was presented as an abstract at ACC Scientific Sessions, April 2–4, 2022.

### Acknowledgments

The sponsors of the trial were Boehringer Ingelheim and Eli Lilly and Company. Boehringer Ingelheim had the organizational oversight over the EMPULSE trial, which included trial conduct, supervision, and monitoring of the enrolling study centers, data collection, and storage as well as data analysis. The trial design was developed by the academic members of the Executive Committee in cooperation with representatives from Boehringer Ingelheim, who were also represented in the Executive Committee of the trial. The Executive Committee of EMPULSE, consisting of academic members and representatives of Boehringer Ingelheim, developed the protocol and provided oversight of the trial’s conduct together with the trial sponsor. The sponsor performed statistical analyses of the trial according to a prespecified statistical analysis plan with oversight by the Executive Committee. An independent data and safety monitoring committee reviewed the safety data. Figure support was provided by Michael Trim at 7.4 Limited, Bollington, Cheshire, United Kingdom, and supported financially by Boehringer Ingelheim. General administrative support in relation to development of the final submission package, supported financially by Boehringer Ingelheim, was provided by Charlie Bellinger, Elevate Scientific Solutions, Horsham, West Sussex, United Kingdom.

### Sources of Funding

The EMPULSE trial was funded by the Boehringer Ingelheim and Eli Lilly and Company Diabetes Alliance.

### Disclosures

Dr Kosiborod has received research grants from AstraZeneca and Boehringer Ingelheim, and has served as a consultant for Alnylam, AstraZeneca, Amgen, Applied Therapeutics, Bayer, Boehringer Ingelheim, Cytokinetics, Eli Lilly, Esperion Therapeutics, Janssen, Merck (Diabetes and Cardiovascular), Novo Nordisk, Pharmacosmos, Sanofi, and Vifor. Dr Angermann has received research support from or has been a consultant for Abbott, Boehringer Ingelheim, Medtronic, Novartis, ResMed, Thermo Fisher, Vifor, and the German Federal Ministry of Education and Research. Dr Collins is a consultant for Aiphia, Siemens, Bristol Myers Squibb, Boehringer Ingelheim, and Vixiar and receives research support from the National Institutes of Health, Patient-Centered Outcomes Research Institute, AstraZeneca, and Beckman Coulter. Dr Teerlink has received research support and/or has been a consultant for Amgen, AstraZeneca, Bayer AG, Boehringer Ingelheim, Bristol Myers Squibb, Cytokinetics, Medtronic, Merck, Novartis, Servier, and Windtree Therapeutics. Dr Ponikowski reports personal fees from Boehringer Ingelheim, AstraZeneca, Servier, Bristol Myers Squibb, Amgen, Novartis, Merck, Pfizer, and Berlin Chemie, and grants and personal fees from Vifor Pharma. Dr Comin-Colet has received unrestricted grants from Vifor and Novartis paid directly to his institute, and consulting fees from AstraZeneca, Bayer, and Boehringer Ingelheim. Dr Ferreira is a consultant for Boehringer Ingelheim and receives research support from AstraZeneca. Dr Mentz reports research support and personal fees from Boehringer Ingelheim, Abbott, American Regent, Amgen, AstraZeneca, Bayer, Boston Scientific, Cytokinetics, Fast BioMedical, Gilead, Innolife, Medtronic, Merck, Novartis, Relypsa, Respicardia, Roche, Sanofi, Vifor, Windtree Therapeutics, and Zoll. Dr Nassif has received speaking honoraria from Abbott and is a consultant for Vifor, Roche, and Amgen. Dr Tromp is supported by the National University of Singapore Start-Up grant; has been a consultant for and holds minor stocks in Us2.ai; has received personal fees from Roche Diagnostics, Daiichi Sankyo, and Boehringer Ingelheim; and has a patent awarded for an “automatic clinical workflow” that recognizes and analyzes 2-dimensional and Doppler modality echocardiogram images for automated cardiac measurements. Dr Brueckmann and Dr Salsali are employees of Boehringer Ingelheim. J.P. Blatchford is an employee of Elderbrook Solutions GmbH. Dr Voors has received research support or has been a consultant for Amgen, AstraZeneca, Bayer AG, Boehringer Ingelheim, Cytokinetics, Merck, Myokardia, Novo Nordisk, Novartis, and Roche Diagnostics. Drs Biegus and Psotka report no conflicts.

### Supplemental Material

Figure S1

## Supplementary Material


