# Supplementary material for: Effects of Empagliflozin on Symptoms, Physical Limitations, and Quality of Life in Patients Hospitalized for Acute Heart Failure: Results From the EMPULSE Trial
Source: Circulation. 2022 Apr 4;146(4):279–88. doi: 10.1161/CIRCULATIONAHA.122.059725 (PMC9311476; doi:10.1161/CIRCULATIONAHA.122.059725)
Supplement: Supplementary file 1 [file cir-146-279-s001.pdf]

SUPPLEMENTAL MATERIAL

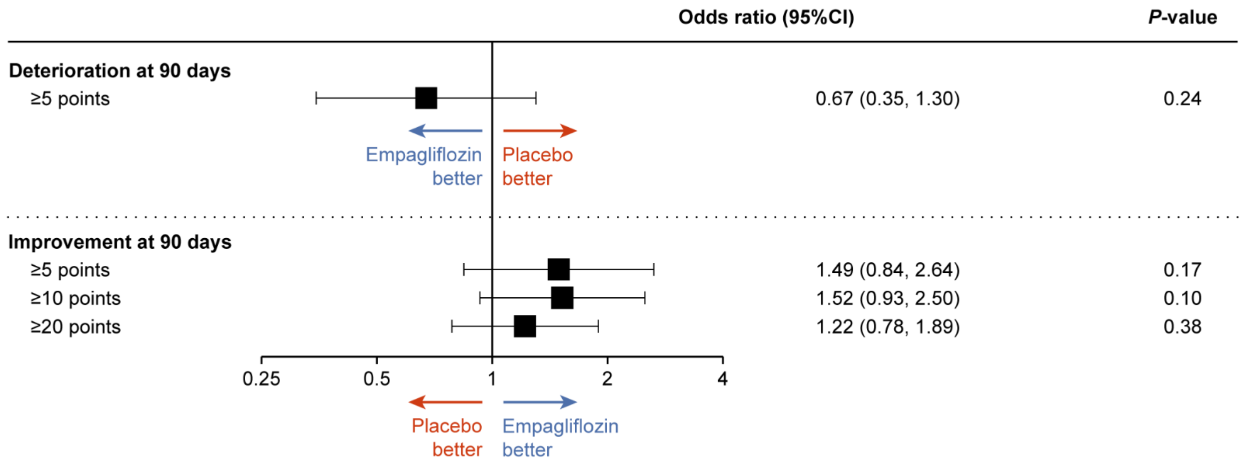

Figure S1. Responder analysis for KCCQ-TSS deterioration and improvements at Day 90. CI, confidence interval; KCCQ-TSS, Kansas City Cardiomyopathy Questionnaire-total symptom score.
